# Taste Masked Orally Disintegrating Pellets of Antihistaminic and Mucolytic Drug: Formulation, Characterization, and *In Vivo* Studies in Human

**DOI:** 10.1155/2014/504536

**Published:** 2014-10-28

**Authors:** Yasmeen Taj, Roopa S. Pai, V. Kusum Devi, Gurinder Singh

**Affiliations:** Department of Pharmaceutics, Faculty of Pharmacy, Al-Ameen College of Pharmacy, Lal Bagh Main gate, Hosur Road, Bangalore, Karnataka 560027, India

## Abstract

The main aim of the present study was to evaluate the potential of orally disintegrating pellets (ODPs) as an approach for taste masking of bitter drugs, namely, Ambroxol hydrochloride (A-HCl) and Cetirizine dihydrochloride (C-DHCl). Pellets were prepared by extrusion/spheronization with Eudragit EPO, kyron T-134, Kyron T-314, mannitol, sorbitol, MCC (Avicel PH-101), sucralose, chocolate flavor, and 5% xanthum gum. The prepared pellets were characterized for percentage yield, drug content, particle size, *in vitro* drug release, and *in vivo* evaluation on humans for taste, mouth feel, and *in vivo* disintegration time. The results revealed that the average size of pellets was influenced greatly by the percentage of binder and extrusion speed. The optimized ODPs disintegrated in less than 20 s and showed more than 98% of drugs in ODPs dissolved within 15 min. Taste perception study was carried out on human volunteers to evaluate the taste masking ability of ODPs for taste, mouth feel, and *in vivo* disintegration time. Crystalline state evaluation of drugs in the optimized ODPs was conducted for X-ray powder diffraction. In conclusion, the study confirmed that ODPs can be utilized as an alternative approach for effective taste masking and rapid disintegration in the oral cavity.

## 1. Introduction

Pharmaceutical science and technology has progressed enormously in the recent years. This increased awareness and has resulted in an increased sophistication and level of expertise in the design, development, manufacture, testing, and regulation of drugs and dosage forms [1]. Today, great importance is given to the organoleptic characteristics of pharmaceutical products mainly appearance, odour, and taste. Customer satisfaction for pharmaceutical products is largely dependent on visual appearance, taste, and textural properties [2]. Any drug has beneficial effect when it is accepted and taken properly by the patient where flavor, fragrance, and colour in a pharmaceutical product contribute to its acceptance. Taste, smell, texture, and aftertaste are important factors in the development of oral dosage forms and establishment of parameters for governing paediatric patient compliance [3].

Undesirable taste is one of several significant formulation problems that are encountered with certain drugs, and masking of the unpleasant taste of a drug improves the product value [3]. With respect to patient acceptability and compliance, taste is one of the prime factors determining the market penetration and commercial success of oral formulations, especially in pediatric medicine [4]. Hence, pharmaceutical industries invest time, money, and resources into developing palatable and pleasant tasting products because good tasting products not only enhance the patient compliance but also provide a competitive advantage. When a therapeutic category is crowded with similar products (e.g., anti-infectives) and provides brand recognition to combat private-label competition as consumers will choose the brand that has the least objectionable taste. Thus, in the present days, taste masking of bitter agents in the pharmaceutical industry has become commercially motivated activity for huge success of the product [5].

Pellets have gained interest as an oral multiple-unit dosage form because they provide reproducible bioavailability and lowered risk of side effects due to dose dumping [6]. Pellets also offer technological advantages such as better flow properties, less friable dosage form, narrow particle size distribution, and uniform packing. The uniform dispersion of a drug into small dosage units reduces the risk of high local drug concentration and their potentially irritating effect on gastric mucosa. Pelletization is an agglomeration process that converts fine powders or granules of bulk drugs and excipients into small, free-flowing spherical or semispherical units, referred to as pellets [7].

A-HCl is used in the treatment of bronchitis and C-DHCl is used in the treatment of allergic rhinitis with asthma. Both drugs are slightly soluble in water and have extremely bitter taste. Their taste masked formulations are available in doses of 30 mg for A-HCL and 5 and 10 mg for C-DHCL as tablets. Hence, masking the taste and improving the rate of dissolution becomes important consideration. These marketed formulations suffer from the major drawback of unacceptable taste. The novelty of this work lies in the application of ODPs (comprising drugs, taste masking agent, and superdisintegrants) as an approach for taste masking and rapid disintegration. To the best of our knowledge, this is the first complete study to formulate and evaluate taste masked ODPs of these molecules in combination.

## 2. Materials and Methods

### 2.1. Materials

Ambroxol hydrochloride was provided ex-gratis by M/s Shilpa Medicare limited, Raichur, India. Cetirizine dihydrochloride was received as a gift sample from M/s Lincoln Pharmaceuticals, Gujarat, India. Eudragit EPO was kindly donated by M/s Dr. Reddy's Laboratories, Hyderabad, India. Kyron T-134 and kyron T-314 were received as a gift sample from M/s Corel Pharma Chem, Ahmedabad, Gujarat, India. Sorbitol, mannitol, and sucralose were acquired from M/S Himedia Laboratories Pvt. Ltd, Mumbai. Microcrystalline cellulose (Avicel PH-101) was supplied by M/s S.D fine chemicals limited, Mumbai. Other reagents used were of analytical grade.

### 2.2. Selection of Binder for Pellets

In preliminary work, binder selection was carried out using polyvinyl pyrrolidone K30 (PVP K30) and sodium carboxy methyl cellulose (Sodium CMC (low viscosity grade)). Depending on the visual inspection of the pellets obtained from the initial studies, it was observed that PVP K30, as a binder, gave acceptable spherical pellets with good yield and low friability, when compared to Sodium CMC. Hence, PVP K30 was selected based on its capability to produce spherical shaped pellets. Concentration of selected binder PVP K30 was optimized by different trials with different ratios, namely, 3%, 4%, and 5%. Based on the observations, 5% of PVP K30 was selected for further formulations [8].

### 2.3. Preparation of Drug-Loaded ODPs

Pellets were prepared by laboratory scale extrusion/spheronization process by following steps.

#### 2.3.1. Preparation of Wet Mass

The wet mass was produced by dry mixing the powders for 5 min in a blender mixer (Hobart, London, UK). A powder mixture constituted A-HCl, C-DHCl, kyron T-134 (polacrillin potassium), Eudragit EPO, mannitol, sorbitol, MCC, kyron T-314, sucralose, chocolate flavor, and xanthum gum (Table 1). Purified water was used as the binder liquid containing PVP K-30 at a concentration of 5%. A precisely determined amount of binder liquid (30 mL/100 g) was then added to the powder mixture and the mass was kneaded for a preset period of time after the liquid addition. The pellets were prepared under standardized operating conditions [6, 7].

#### 2.3.2. Extrusion

The wet powder mass was immediately extruded at 75 rpm through a radial screen extruder (Model S250, Umang Pharmascitech, Mumbai, India), supplied with a 1 mm aperture screen [6, 7].

#### 2.3.3. Spheronization

A radial plate spheronizer with a plate diameter of 45.0 cm was used (model S250, Umang Pharmascitech, Mumbai, India). The friction plate speed in the spheronizer was kept at 1000 rpm for 15–20 min. The wet pellets were dried in a hot air oven at 40°C for 24 hours and then stored in sealed bags.

### 2.4. Characterization of ODPs

The pellets of the different formulations were characterized for the following properties.

#### 2.4.1. Size

Pellet size was evaluated with a Motic BA300 microscope (China) connected to a video camera. Size was estimated by Motic images plus 2.0 software for a total of 100 pellets per lot. The size data were best fitted by a normal distribution [9]. In addition, photomicrographs of pellets were obtained with a scanning electron microscope (Jeol JSM 5600 LV, Jeol, Tokyo, Japan).

#### 2.4.2. Flow Properties

*(1) Particle Size Distribution.* The pellet size and pellet size distribution were estimated by sieve analysis (Test sieve shaker, Retsch VE1000 shaker, Germany) and optical microscope. Each batch of the pellets was sieved before the subsequent* t*-test in order to remove the lumps larger than 1000 *μ*m. About 20 g of the sample was sieved using a set of standard sieves (1600, 1250, 1000, 800, 710, 630, 500, and 400 *μ*m) trembling at an amplitude of 2 mm for 5 min. The fraction remaining on each screen was weighed and expressed as percentage of the total weight and medium diameter of pellets was obtained according to the cumulative size distribution. In optical microscopy method, particle size of pellets was determined by using 10x objective lens. A clean glass slide was taken and small drop of glycerine was added and spread over the slide. Small quantities of pellets were then placed over the slide and particle size of about 100 pellets was determined [6, 7]. All results presented were the mean of three determinations. Pellets ranging between 850 and 1000 *μ*m in size were selected for* in vitro* dissolution test so that the effect of particle size on dissolution rate is excluded.

*(2) Angle of Repose.* The static angle of repose was measured according to the standard reported method. Angle of repose (*θ*°) was calculated from the standard trigonometric relationship [10].

(*3) Volume, Density, and Compressibility.* 20 g sample was put into a 200 mL graduated cylinder of a volume and density apparatus (Electrolab TAB density Tester, USP, Model ETD 1020, Mumbai). Bulk density, tapped density, and Carr's percent compressibility were calculated [11, 12].

*(4) Assay of the Drug Content.* The samples were assayed by a UV spectroscopic method according to the Indian Pharmacopoeial 2007 norms. ODPs equivalent to 30 mg of A-HCl and 5 mg of C-DHCl of the drug were weighed and grounded to fine powder and dissolved in 20 mL of methanol and sonicated for 10 min to dissolve it completely and diluted in 100 mL with distilled water and filtered through Whatman filter paper (0.45 *μ*m). The samples were analyzed simultaneously by spectrophotometrically at a wavelength of 244 nm for A-HCl and 230 nm for C-DHCl (Model UV-1700, UV-Visible spectrophotometer, Shimadzu, Kyoto, Japan).

*(5) In Vitro Disintegration Time*. The* in vitro* disintegration time was determined for the pellets. This test was performed to ensure disintegration of pellets in the salivary fluid.* In vitro* disintegration time was measured by dropping a little quantity of the pellets in a measuring cylinder containing 6 mL of simulated salivary fluid of pH 6.8. The disintegration time was defined as the time necessary for the ODPs to completely disintegrate until no solid residue remains or only a trace amount of soft residue remains on the screen. A digital stopwatch was used to record the disintegration time to the nearest second. Only one ODPs was analyzed at a time in order to ensure maximum accuracy. All results are presented as mean value ± SD (*n* = 6).

### 2.5. Taste Perception Study and Statistical Analysis

Taste perception was carried out on a previously consented group of 6 healthy human volunteers. The protocol for the investigation was approved by institutional animal and human ethics committee (IAHEC) and informed consent was signed by the volunteers before starting the study. Prior to the test, all volunteers were asked to rinse their mouth with portable drinking water. To find a suitable amount of drug for the evaluation of the bitter taste intensity during the comparative test, the perception and bitterness recognition threshold of capsules containing ODPs formulation (P4) and marketed formulation were evaluated. The determination of the threshold was carried out as follows. The volunteers were asked to taste samples (2.5 mg) kept in the mouth for 60 seconds and then they spat out. Then, the volunteers were asked for the following perceptions:

1. “I feel bitter taste.”
2. “I feel something but I cannot identify the taste.”
3. “I do not feel any taste.”

For comparative analysis, volunteers were made to taste the samples and rate them on a hedonic rating scale as per the bitterness of the samples (ranging from “excellent” for no taste perception and “extremely poor” for bitter taste). The average rating score was then used to categorize the sample. Finally, the roughness levels were recorded on a numerical scale ranging from 0 to 3, where 0, 1, 2, and 3 indicate no, slight, moderate, and high roughness, respectively. After tasting the sample, subjects rinsed their mouth with water completely. The obtained results were subjected for statistical analysis using one way ANOVA followed by Dunnetts multiple comparison tests. The values obtained were expressed as Mean ± SEM [13].

### 2.6. *In Vitro* Dissolution Studies

Dissolution studies were carried out following the USP II paddle method at 37°C and 75 rpm using a dissolution tester (Electrolab TDT-06PL Dissolution tester, Mumbai, India). The dissolution medium was 900 mL simulated saliva fluid without enzymes (SSF) at pH = 6.8. The amount of ODPs used was equivalent to 30 mg of A-HCl and 5 mg of C-DHCl. At specified time intervals (1, 5, 10, 15, 20, 25, and 30 mins), samples were withdrawn, filtered through 0.45 *μ*m millipore filter, and assayed simultaneously for drug content spectrophotometrically at 244 nm for A-HCl and 230 nm for C-DHCl against blank after appropriate dilution.

### 2.7. Preparation of Capsules Containing ODPS

Based on the drug content,* in vitro* disintegration, and* in vitro* drug release studies, formulation P4 was selected as the best formulation for ODPs. The pellets were filled into body of capsule of size 2. The total dose of the drug in capsule was amounting up to 30 mg for A-HCl and 5 mg of C-DHCl.

### 2.8. *In Vitro* Release Studies of Capsule Containing Formulation (P4)

The drug release profile of filled capsules was determined using USP apparatus of basket type at 37 ± 0.5°C and rotational speed of 75 rpm in 900 mL of simulated salivary fluid pH 6.8 solution. 5 mL of dissolution medium was withdrawn at intervals of 5 min for 45 min and then filtered through Whatman filter paper. Withdrawn samples were simultaneously analyzed spectrophotometrically at 244 nm for A-HCl and 230 nm for C-DHCl against blank in Shimadzu U.V. spectrophotometer and absorbance was recorded.

### 2.9. X-Ray Diffraction Pattern

XRD studies of A-HCl, C-DHCl, and capsule containing formulation P4 were carried out using an X-ray diffractometer (Brucker AXS, D8 advanced, Germany) with monochromatic CuK*α* radiation ($\lambda =1.5407\overset{´}{Å}$), voltage 50 kV, current 100 mA, and 2*θ* over a 2–45° range.

### 2.10. Drug Release Studies of Optimized ODPs vis-à-vis Other Products

Drug release profiles of the optimized ODPs were compared with those of the conventional marketed brand and pure drug, each containing 30 mg of A-HCl and 5 mg of C-DHCl.

## 3. Results and Discussion

A-HCl is a secretolytic agent used in the treatment of respiratory diseases associated with viscid or excessive mucus. C-DHCl is a potent H_1_ receptor antagonist without any significant anticholinergic and antiserotonin effects. An ideal combination of C-DHCl with A-HCl is prescribed for paediatrics which provides immediate relief from allergic conditions, like rhinitis, sinusitis, allergic cough, nasal congestion, and itching. These two drugs are very bitter in taste and practically slightly soluble in water and, hence, were selected for taste masking and formulating into orally disintegrating solid dosage form.

A powder mixture constituted A-HCl and C-DHCl, kyron T-134 (polacrillin potassium), Eudragit EPO, mannitol, sorbitol, MCC, kyron T-314, sucralose, chocolate flavor, and xanthum gum were the composition of the pellets prepared by extrusion/spheronization. The type and amount of granulation liquid was selected by investigating water, isopropyl alcohol, and combination of water and isopropyl alcohol in different ratios. After several trials with different batches of pellets, purified water was selected as wet massing liquid.

The optimum level of granulating fluid is that level which results in maximum pellet roundness in the targeted particle size range. In preliminary work, every formulation was granulated using a series of water levels. The optimum level was identified as that level which produced nearly round particles whose average size is similar to the aperture of the extrusion screen. Binder selection was carried out using PVP K30 and Sodium CMC. PVP K30 was selected based on its capability to produce spherical shaped pellets. Concentration of selected binder was optimized to be 5%.

The amount and concentration of binder affected the appearance of the resulting pellets. Increasing the volume of binder solution augmented the mean size of pellets but dwindled the yield in the desirable pellet size range. The exploit of an overindulgence amount of binder gave rod shaped pellets and increased the hardness of the pellets. Due to the influence of the amount of granulating liquid on the processability of the wet mass, the volume of granulating liquid was further optimized to achieve accurate wetting of mass.

An increase in spheronization speed has been already reported as beneficial for pellet sphericity. This was arbitrated in terms of a high batch yield and a high percentage of pellets obtained with size ranging from 700 to 850 *μ*m. At higher water concentrations, agglomeration occurred, whereas using a suboptimal amount of water resulted in dumbbells. The significant interaction between water and spheronization speed indicated that a low spheronization speed and water level decreased the sphericity of the end product.

During the formulation, different superdisintegrants SSG and kyron T-314 (polacrillin potassium), different diluents (mannitol and mannitol with sorbitol) were screened. It was seen that formulation containing kyron T-314 (polacrillin potassium) as superdisintegrant showed better results compared to SSG; thus, it was selected for the further formulation. The prepared batches were coded as P1, P2, P3, and P4.

### 3.1. Pellets Size and Shape

In general, the volume weighted mean of the manufactured pellets was found to be in a range from 720 to 740 *μ*m and is shown in Table 2. In addition, the particle size distribution of ODPs is characterized by small span values. Calculated span value for all ODPs formulations was found to be 0.59–0.67, indicating a narrow particle size distribution.

### 3.2. Angle of Repose and Drug Content

The angle of repose and flow rates of ODPs formulations were carried out. It was observed that the ODPs formulations had minimum angle of repose and good circularity as well as high density and high flow rate according to fixed funnel method. The compactness of pellets should be taken into consideration not only for technological purposes, but also because the majority of them are typically encapsulated in two-piece hard shell capsules and consequently, for these fixed volume dosage forms, the density will determine the fill weight. The drug content in the ODPs was 85.2 ± 1.2% to 95.4% ± 0.8, indicating the uniformity in drug content Table 2.

### 3.3. *In Vitro* Disintegration Test

*In vitro* disintegration test was carried out in simulated salivary fluid pH 6.8 for 30 min of all the ODPs formulations P1 to P4. All the formulations exhibited disintegration time between 12.33 and 16.67 sec (Table 2).

### 3.4. Taste Perception Studies

In order to evaluate the efficiency of taste masking by ODPs, a taste perception study was designed and followed. Precisely, a hedonic rating scale (Table 3) of 1 to 7 was used for the quantification of the results of taste perception study. The overall ratings for various samples were obtained and the mean rating was used to qualify the taste masking efficiency.

### 3.5. *In Vitro* Dissolution Studies

**Figure 1:**
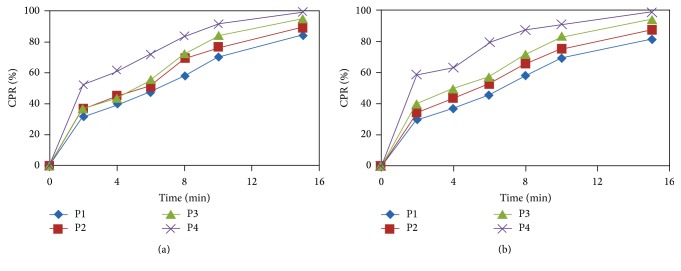
(a) Comparison of* in vitro* dissolution study of taste masked ODPs P1 to P4 containing A-HCl. (b) Comparison of* in vitro* dissolution study of taste masked orally disintegrating pellets P1 to P4 containing C-DHCl.

In the present study, dissolution results for all the ODPs formulations P1–P4 are illustrated in Figures 1(a) and 1(b). The rate and extent of drug dissolution were greatly enhanced from the prepared ODPs formulations compared to the plain drug. Results show maximum dissolution of drug from ODPs formulation P4 containing 140 mg of superdisintegrant kyron T-314. Statistical analysis revealed that only the type of superdisintegrant had a significant effect on the percentage of drug dissolved after 15 min.

**Figure 2:**
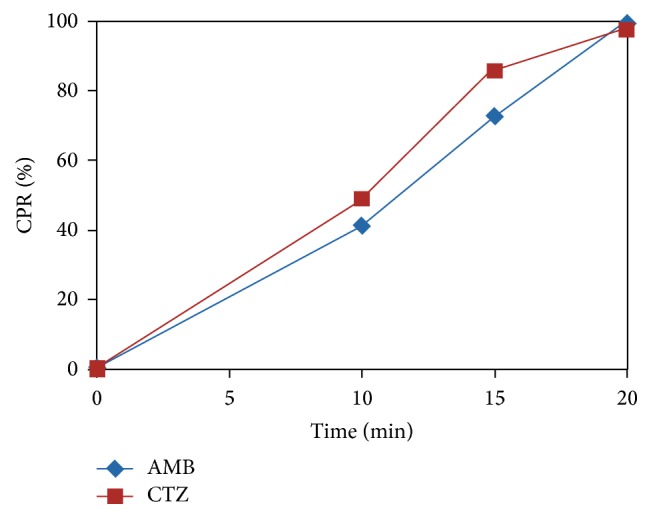
*In vitro* dissolution study of capsules containing taste masked ODPs formulation (P4).

Based on the drug content,* in vitro* disintegration, and* in vitro* drug release studies, ODPs formulation P4 was selected as the best formulation for orally disintegrating pellets. The pellets were filled into body of capsule of size 2. The* in vitro* release study for capsule formulation was carried out in simulated salivary fluid pH 6.8, for 20 min as portrayed in Figure 2. Results exhibited a release of 99.4% for A-HCL and 97.8% for C-DHCL at the end of the 20 min.

### 3.6. *In Vitro* Drug Release Comparison with Marketed Formulation

**Figure 3:**
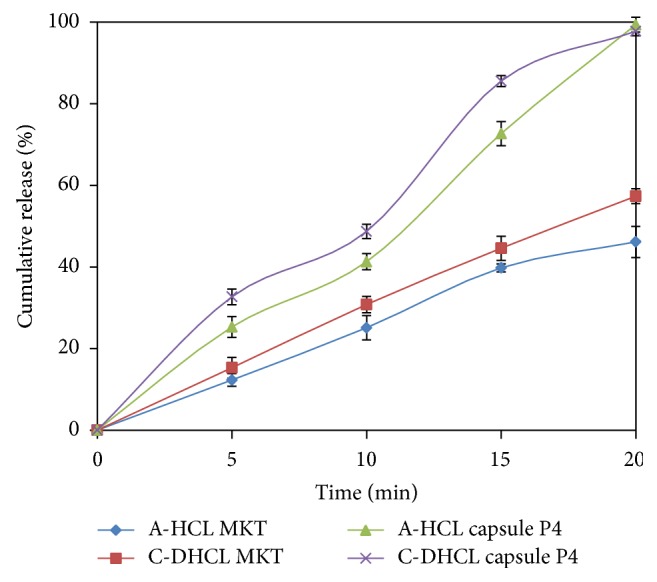
*In vitro* drug release comparison with marketed formulation.

Marked improvement was observed in the drug release profiles (Figure 3) of the optimized tasted masked ODPs formulation P4 as compared to that of marketed formulation and pure drug. Drug dissolution was nearly completed within 20 min in case of the optimized formulation P4, as compared to that of the MKT and pure drug, wherein drug release was only 39.8% for A-HCl and 44.8% for C-DHCl in pH 6.8 at the end of 15 min, where optimized ODPs formulation P4 exhibited a release of 99.5% for A-HCl and 98.4% for C-DHCl at the end of the 15 min. Analogous* in vitro* dissolution profile of optimized ODPs formulation P4 construe that the superdisintegrant seem to affect the drug release from the ODPs significantly.

### 3.7. Powder X-Ray Diffraction (XRD)

**Figure 4:**
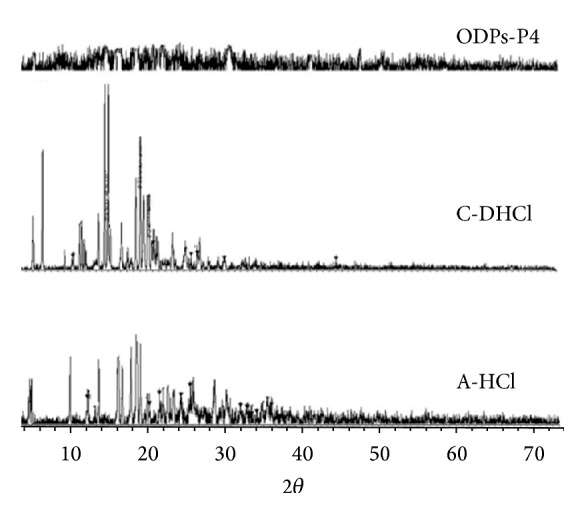
Comparison of powder X-ray diffractometry of A-HCl, C-DHCl, and ODPs formulation P4.

Figure 4 shows the powder XRD pattern of A-HCl and C-DHCl plain powder and the optimized tasted masked ODPs formulation P4. The crystalline nature of A-HCl and C-DHCl powder is exhibited by a strong and characteristic XRD pattern. A-HCl and C-DHCl shows intense scattering peaks located at 10.03°, 13.34°, 17.62°, 20.6°, and 2*θ*. The diffraction pattern of the physical mixture of the drugs and excipients showed the peaks corresponding to the crystalline drug molecules present in the mixture. The diffraction pattern in optimized tasted masked ODPs showed absence, broadening, and reduction of major A-HCl and C-DHCl diffraction peaks indicating that mostly an amorphous form (disordered state) existed in the ODPs.

### 3.8. Statistical Analysis

Disintegration time, sensory evaluation of roughness, and taste evaluation in mouth of adult healthy human volunteers were carried out for capsule containing orally disintegrating pellets P4 (CCTMDPs) along with the Conventional marketed formulation (CMF). Complete masking of the bitter taste was observed in all the formulations, when compared to that of the marketed formulation are presented in Tables 4 and 5.

**Figure 5:**
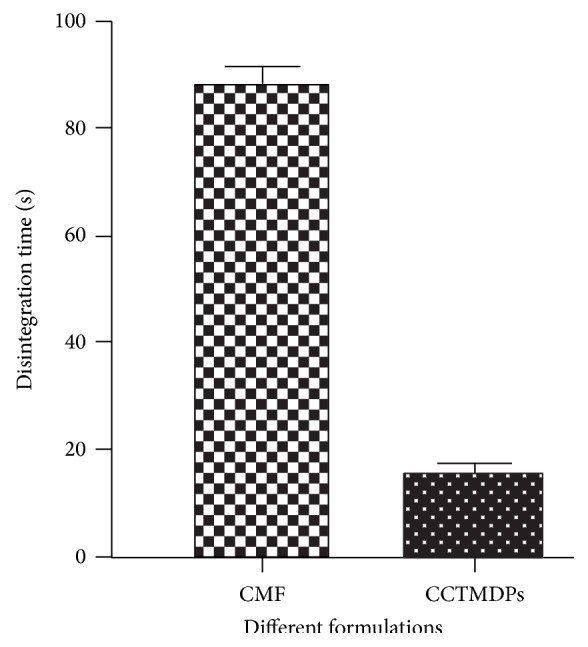
Comparison of mean disintegration time of different formulations for statistical analysis.

The obtained results were subjected for statistical analysis using one way ANOVA followed by Dunnetts multiple comparison tests [14]. The values obtained were expressed as Mean ± SEM and are portrayed in Figure 5. In all the three groups, the results were significant (*P* < 0.05) and were obtained when compared to the conventional marketed formulation. Extremely significant results (*P* < 0.001) were observed for capsule containing taste masked disintegrating pellets (CCTMDPs). Hence, CCTMDPs were found to be better than the conventional formulations.

## 4. Conclusions

A promising formulation of a ODPs of Ambroxol hydrochloride and Cetirizine dihydrochloride made of superdisintegrants is successfully prepared by extrusion/spheronization. The drug loaded pellets exhibited very less disintegration time and excellent drug release in 15 min. The study suggests that the optimized formulation developed in this work may be an alternative to conventional formulations of Ambroxol hydrochloride and Cetirizine dihydrochloride. In future, the orally disintegrating solid dosage forms may be most acceptable and prescribed dosage form due to its quick action. Thus, the “patient-friendly dosage form” of the bitter drugs, especially for paediatric, geriatric, bedridden and noncooperative patients was successfully developed using these technologies.

## Figures and Tables

**Table 1:** Formulation design for ODPs containing A-HCL and C-DHCL.

Composition (mg)	P1	P2	P3	P4
Ambroxol hydrochloride	3	30	30	30
Cetirizine Dihydrochloride	5	5	5	5
Kyron T-134 (polacrillin potassium)	120	70	—	140
Eudragit EPO	20	70	140	—
Mannitol	55.24	55.24	55.24	55.24
Sorbitol	27.66	27.66	27.66	27.66
MCC	30	30	30	30
Kyron T-314	12	12	12	12
Sucralose	0.2	0.2	0.2	0.2
Chocolate flavor	q.s	q.s	q.s	q.s
Xanthum gum	5%	5%	5%	5%

**Table 2:** Angle of repose, percentage of drug content, and disintegration time of taste masked ODPs formulations P1–P4.

PF-code	Particle size (*μ*m)	Span value	AOR	Drug content (%)		DT (sec)
				A-HCl	C-DHCl	
P1	736	0.62	24.72 ± 1.67	93.82 ± 0.40	90.92 ± 0.257	14.33 ± 0.57
P2	729	0.59	26.08 ± 2.53	94.91 ± 0.20	93.01 ± 0.344	16.67 ± 0.56
P3	723	0.67	22.62 ± 2.40	96.18 ± 0.26	97.22 ± 0.301	15.00 ± 0.0
P4	728	0.63	25.19 ± 1.88	98.09 ± .146	98.18 ± 0.136	12.33 ± 0.57

PF = pellet formulation, AOR = angle of repose, and DT = disintegration time.

**Table 3:** Hedonic rating scale for taste perception study.

Rating	Description
7	Excellent
6	Very good
5	Good
4	Fair
3	Poor
2	Very poor
1	Extremely poor

Ratings were used to categorized the taste perception of the samples ranging from 1 = extremely poor to 7 = excellent.

**Table 4:** Statistical analysis for evaluation of disintegration time in mouth of healthy adult human volunteers.

Sl. No.	Groups (*n* = 6)	Mean disintegration time ± SEM
1.	Conventional marketed formulation (CMF)	87.8 ± 3.26
2.	Capsule containing taste masked disintegrating pellets (CCTMDPs)	14.5 ± 2.32^***^

*P* value < 0.05 = ^*^significant; *P* value < 0.01 = ^**^highly significant; *P* value < 0.001 = ^***^extremely significant.

**Table 5:** Statistical analysis of disintegration time in mouth of healthy adult human volunteers by Dunnett's multiple comparison tTest.

Sl. No.	Dunnett's multiple comparison test	Significant?	Summary
		*P* < 0.05?	
1	CMF vs CCTMDP	Yes	∗∗∗
